# The Hodgkin Lymphoma Microenvironment: Insights from Spatial Transcriptomics

**DOI:** 10.3390/ijms27083689

**Published:** 2026-04-21

**Authors:** Ruth Alonso-Alonso, Victoria Menendez, Eva M. Vázquez, José L. Solórzano, Juan F. García

**Affiliations:** 1 Translational Research, MD Anderson Cancer Center Foundation, 28033 Madrid, Spain; r.alonsoa@hospiten.es; 2 uc3m—Santander Big Data Institute (IBiDat), 28903 Getafe, Spain; victoriamdanderson@gmail.com; 3 Pathology and Molecular Diagnostic Department, MD Anderson Cancer Center, 28033 Madrid, Spain; e.vazquez@mdanderson.es (E.M.V.); jlsolorzano@mdanderson.es (J.L.S.)

**Keywords:** spatial transcriptomics, classical Hodgkin lymphoma, tumor microenvironment, digital spatial profiling, Hodgkin Reed–Sternberg (HRS) cells, cell–cell interactions, immune evasion

## Abstract

Classical Hodgkin lymphoma (cHL) is a paradigmatic example of a malignancy in which the tumor microenvironment (TME) plays a dominant role in disease biology. Malignant Hodgkin and Reed–Sternberg (HRS) cells typically constitute only a small minority of the tumor mass (approximately 1–5%). HRS cells are embedded within a complex, highly structured immune and stromal milieu that drives survival, immune evasion, and therapy response. Over the past decade, transcriptomic approaches, particularly single-cell RNA sequencing, have reshaped our understanding of cellular heterogeneity within cHL. However, these approaches lack spatial context, a limitation that is especially relevant in cHL, where cell–cell interactions and physical proximity are central to immune evasion and tumor support. Recent advances in spatial transcriptomics now enable genome-scale, spatially resolved interrogation of gene expression in situ. In this review, we summarize spatially resolved studies of the cHL microenvironment, discuss what they reveal about HRS-centered cellular niches and immune evasion, and highlight how these findings may inform risk stratification, biomarker discovery and microenvironment-directed therapies.

## 1. Introduction: Why Microenvironment Matters in Hodgkin Lymphoma

Classical Hodgkin lymphoma (cHL) is a common lymphoma, with more than 80,000 new cases and approximately 25,000 deaths reported worldwide each year [1]. Despite this, it represents one of the most curable hematologic malignancies, with long-term survival rates exceeding 80% in high-income settings; however, a subset of patients—particularly those with advanced stages or primary refractory disease—continue to experience poor outcomes [2,3]. Clinically, cHL arises in lymph nodes, particularly in cervical, mediastinal, and axillary regions [2,4], where malignant Hodgkin and Reed–Sternberg (HRS) cells reside within a highly reactive immune and stromal microenvironment [2,5]. Patients typically present with painless lymphadenopathy and may develop systemic “B symptoms,” including fever, night sweats, and weight loss, as well as fatigue or pruritus [2,4].

cHL is distinct from non-classical Hodgkin lymphoma, nodular lymphocyte-predominant Hodgkin lymphoma (NLPHL), which has been recognized as a separate clinicopathological entity. Despite some morphological similarities, there is ample clinicopathological, etiological, and pathogenetic evidence that NLPHL is a different neoplasm from cHL [5,6].

Standard treatment for cHL is stage-adapted and generally based on combination chemotherapy regimens such as ABVD, often combined with radiotherapy in early-stage disease [4]. In the relapsed or refractory setting, targeted therapies—including antibody–drug conjugates such as brentuximab vedotin and immune checkpoint inhibitors targeting PD-1—have significantly improved outcomes [7].

cHL is characterized by rare malignant HRS cells embedded within a dense immune infiltrate, including T lymphocytes, B cells, macrophages, eosinophils, and other immune and stromal components. This tumor microenvironment (TME) is central to disease biology and actively shapes tumor cell survival, immune escape, and therapeutic response [5,8].

For decades, histopathology and immunohistochemistry (IHC) suggested that these non-malignant cells are not passive bystanders but active contributors to tumor growth, immune evasion, and treatment response. HRS cells sculpt their surroundings through cytokines and chemokines such as CCL17/TARC and CCL22, which recruit and retain CD4+ T-cell populations, and through immunomodulatory pathways that promote local T-cell dysfunction. Immune checkpoint axes—including PD-1/PD-L1, CTLA-4, LAG-3, TIM-3, TIGIT, and CD137/CD137L—together with myeloid- and stromal-derived suppressive signals, provide a biological rationale for immune checkpoint blockade, which has demonstrated clinical activity in relapsed or refractory cHL [9,10,11].

Earlier studies analyzed the overall abundance or frequencies of specific immune populations in the TME using single-marker IHC or limited marker panels and reported correlations with clinical course and therapeutic response. These observations laid the foundations for current pathogenesis models and reinforced the view that tumor-induced TME features can act as outcome predictors, beyond a mere reflection of an ineffective immune response.

However, the clinical relevance of immune components in cHL TME is not straightforward, and there is growing evidence of substantial phenotypic complexity and plasticity. Although these narrow-panel approaches linked certain immune-cell frequencies to outcome, their clinical applicability has been limited by technical standardization issues and cohort-to-cohort variability. In addition, spatial heterogeneity likely contributes to interobserver variability and limited reproducibility across cohorts [5,12].

While bulk transcriptomics and, more recently, single-cell RNA sequencing (scRNA-seq) have provided detailed cellular and molecular inventories of the cHL microenvironment, they do not preserve information on spatial organization. In cHL, where HRS cells rely on direct and short-range interactions with surrounding immune cells, spatial context is critical.

Spatial transcriptomics has emerged as an approach that complements single-cell methods by preserving tissue architecture while capturing gene expression at increasingly high resolution, enabling the study of spatially defined neighborhoods, proximity-dependent immune states, and tumor–microenvironment interactions in situ [13,14].

In this review, we summarize recent spatial studies of the cHL microenvironment (2020–2026), highlighting how spatial organization refines non-spatial models of cHL biology and discussing translational opportunities for implementing spatial biomarkers and microenvironment-directed therapies in cHL.

## 2. Spatial Profiling Technologies for Studying the cHL Microenvironment

Spatial transcriptomics refers to a range of technologies that map gene expression to defined locations within tissue sections. In broad terms, these platforms aim to overcome the main limitation of conventional bulk transcriptomics—signal averaging across heterogeneous cell populations and loss of positional information—while extending classical in situ approaches (IHC/ISH), which preserve architecture but are typically limited in multiplexing depth [13]. In cHL, these advantages are especially relevant because rare HRS cells exist within highly organized immune and stromal niches, where proximity-dependent interactions are central to tumor support and immune evasion.

### 2.1. Sequencing-Based, Transcriptome-Wide Platforms

Sequencing-based spatial transcriptomics captures transcriptome-wide RNA profiles using spatially barcoded capture arrays. Platforms such as 10× Genomics Visium and related methods (e.g., Slide-seq and high-definition spatial transcriptomics, HDST) enable unbiased identification of spatial gene-expression patterns across tissue sections. Because each capture spot (or spatial unit) can contain transcripts from multiple cells, cell-type assignment often requires integration with scRNA-seq references and/or computational deconvolution to estimate cell-type composition and transcriptional state within each spatial unit [14,15,16,17].

Slide-seq and related approaches map gene expression directly onto tissue sections with near-cellular resolution by using spatially barcoded beads [15]. In the original method, a thin tissue slice is placed onto a surface densely coated with microscopic beads, each carrying a unique DNA barcode that encodes its exact spatial position. Messenger RNA from the tissue binds to nearby beads, capturing transcripts along with their positional barcodes; sequencing then reveals which genes were expressed and where they were located in the tissue. Slide-seqV2 improves on the original by optimizing bead chemistry, array preparation, and RNA capture efficiency, resulting in much higher sensitivity, better gene detection per spot, and more accurate spatial maps while keeping the same core principle of spatially barcoded bead arrays [16].

More recently, high-resolution implementations (e.g., Visium HD) increase spatial resolution and improve the ability to interrogate tumor-adjacent niches, while still requiring careful computational strategies for cell-level inference and interpretation [14,18,19]. Visium HD offers broader discovery potential, but places greater demands on sample quality, provides less flexibility in selecting regions after the experiment begins, and its effective resolution depends on the spot size used for analysis (i.e., each spot may capture transcripts from one or multiple cells).

### 2.2. ROI-Based and Imaging-Based Targeted Platforms

Targeted, image-guided approaches are optimized for clinical specimens and hypothesis-driven questions. NanoString GeoMx Digital Spatial Profiling (DSP) quantifies predefined RNA (or protein) panels from user-defined regions of interest (ROIs) and is compatible with formalin-fixed, paraffin-embedded (FFPE) tissues, enabling the analysis of retrospective cohorts and pathology-aligned sampling. These features make GeoMx especially useful for clinically annotated cHL series. Although targeted GeoMx assays sacrifice transcriptome-wide coverage, the platform now also supports whole-transcriptome workflows; however, it remains ROI-based and still requires careful region selection to minimize sampling bias [18]. GeoMx uses barcoded, photocleavable probes that hybridize to predefined RNA targets in tissue sections and, although initially focused on targeted panels, now also supports transcriptome-wide approaches (WTA); however, it remains ROI-based rather than a whole-section discovery platform.

In parallel, high-plex imaging-based in situ technologies (e.g., CosMx, Spatial Molecular Imaging, Xenium, and academic methods such as MERFISH, seqFISH+, and STARmap) localize transcripts at cellular or near-cellular resolution and can be particularly useful for resolving rare cellular interactions and validating spatial circuits directly in tissue. These approaches often rely on predefined or targeted panels, particularly in current commercial workflows, although some imaging-based platforms and academic methods now support transcriptome-scale or whole-transcriptome readouts [20,21,22,23,24]. In cHL, such resolution may be especially valuable for studying HRS cell neighborhoods and short-range immune interactions that are difficult to infer from non-spatial assays.

### 2.3. Spatial Proteomics and Multimodal Profiling

Although not transcriptomic per se, spatial proteomic platforms have also been highly informative in cHL. Several influential studies have leveraged imaging mass cytometry (IMC) and multiplex immunofluorescence (mIF) to quantify checkpoint markers, myeloid programs, and proximity-based features with single-cell resolution [25,26]. Multimodal designs that combine spatial RNA profiling with protein imaging and histopathology can strengthen biological interpretability and facilitate translation into deployable assays, as protein markers are often more readily implemented in clinical workflows [27].

### 2.4. Analytical Workflows and Integration Strategies

Interpretation of spatial data increasingly depends on computational frameworks that link transcriptional state to spatial context. Deconvolution algorithms have gained significant attention, particularly reference-based methods, which can achieve high accuracy in estimating cell-type abundances from mixed signals. One example is CIBERSORTx, which estimates cell-type proportions and can infer cell-type-specific expression programs from bulk or spatial mixtures using reference expression signatures derived from scRNA-seq [28].

Similarly, EcoTyper is a machine learning framework that identifies cell states and ecosystems from bulk, single-cell, and spatially resolved expression data, enabling large-scale profiling of cellular ecosystems. Because studies often use different reference signatures and preprocessing pipelines, deconvolution performance and reproducibility can vary substantially [29].

Crucially, spatial transcriptomic data are increasingly integrated with scRNA-seq reference atlases. This integration allows assignment of cell identities to spatial locations, inference of cell–cell communication (e.g., ligand–receptor analysis), and identification of spatially defined cellular neighborhoods. Beyond CIBERSORTx and EcoTyper, commonly used spatial mapping/deconvolution frameworks include cell2location, RCTD, DestVI, SPOTlight, and Tangram, among others [30,31,32,33,34].

Spatially informed analyses then quantify compartments and neighborhoods (composition, proximity, and co-localization) and can prioritize candidate tumor–immune interactions for mechanistic follow-up. Analysis ecosystems such as Squidpy and Giotto facilitate neighborhood graph construction, enrichment testing, and visualization, while methods such as SPARK-X explicitly model spatial structure for detecting spatially variable genes [35,36,37]. A practical comparison of representative spatial profiling platforms commonly used in recent cHL studies is provided in Table 1.

Because no single platform fully captures both architectural context and cellular resolution, recent progress in cHL has relied on complementary spatial approaches. The following sections therefore focus on what these methods have specifically revealed about cHL biology, immune evasion, and translational opportunities.

## 3. The Hodgkin Lymphoma Microenvironment: Insights from Non-Spatial Approaches

Traditionally, characterization of the cHL TME relied on IHC with relatively simple lineage markers to identify immune populations. Although these early approaches may now appear reductionist, they generated a substantial body of knowledge demonstrating the functional importance of immune responses in shaping tumor biology [5,12]. Classical studies consistently emphasized the central role of T cells, macrophages, and HRS cell-derived cytokines and chemokines that recruit and modulate immune and stromal populations, including CD4^+^ helper and regulatory T cells, eosinophils, macrophages, mast cells, and fibroblastic elements, collectively generating an ineffective immune environment that paradoxically sustains tumor survival.

Among these infiltrating cells, CD4^+^ T lymphocytes represent the most abundant population [38,39,40,41,42]. They are actively attracted by tumor-derived signals such as CCL17/TARC, yet remain functionally ineffective, a phenomenon previously termed “dilution anergy,” in which the high density of non-tumor-specific helper cells diminishes productive antitumor immunity [43,44]. These early observations established the central importance of CD4^+^ T-cell programs in cHL and anticipated later spatial models in which tumor-associated CD4^+^ subsets occupy specialized niches around HRS cells. Although these insights were largely inferred from non-spatial approaches, later work suggested that spatial organization further shapes their function: T follicular helper cells can form physical barriers around HRS cells, restricting access of cytotoxic effectors, while regulatory T cells (Tregs) reinforce immunosuppression through inhibitory cytokine secretion. Immune escape is therefore promoted by several coordinated mechanisms, including CD4^+^ T-cell rosette formation around tumor cells and skewing toward Treg polarization [45,46,47,48].

Macrophages constitute another major and functionally influential component of the TME. Typically polarized toward an M2-like phenotype [41,49], they are recruited and conditioned by tumor-derived factors such as CSF-1, IL-10, and CCL5 [49,50], which promote their survival and alternative activation [51,52]. Rather than mounting effective phagocytic or antigen-presenting responses, these tumor-associated macrophages support immune evasion by expressing inhibitory ligands such as PD-L1 and secreting anti-inflammatory mediators that suppress effector T-cell function. Differentiation of monocytes into anti-inflammatory tumor-supportive macrophages is associated with increased secretion of suppressive cytokines (IL-4, IL-10, IL-13, TGF-β) and downregulation of antigen-presentation and immune synapse molecules such as MHC-II and CD58. These observations anticipated later spatial studies showing that macrophages often contribute to tumor-supportive niches adjacent to HRS cells [53]. High macrophage density has been associated with adverse prognosis [54,55], although conflicting data highlight the limitations of rigid M1/M2 polarization models and underscore the remarkable plasticity of myeloid populations [56,57].

In contrast, cytotoxic immune compartments are sparse and functionally impaired. CD8^+^ T cells are typically scarce and exhibit reduced cytolytic activity, partly due to frequent loss or downregulation of MHC class I on HRS cells [58], which limits antigen recognition. Natural killer (NK) cells are present but functionally restrained by inhibitory cytokines, checkpoint signaling, and suppressive stromal influences. Cytotoxic CD4^+^ T cells, which could theoretically contribute to tumor killing, are rare [38,59,60], reinforcing the global deficiency of effective cytotoxic surveillance. Reports have suggested that higher proportions of cytotoxic T cells have paradoxically been associated with poorer therapeutic response [41,59,61], whereas increased Treg abundance correlates with improved prognosis [46,59,62,63,64], illustrating the complex and sometimes counterintuitive prognostic impact of immune subsets, particularly when spatial organization is not considered.

A related compartmentalization has been described for myeloid-derived suppressor cells (MDSCs) and their subtypes, which show specific topographies. The area surrounding tumor cells is distinctly enriched in m-MDSCs and T cells with an exhausted phenotype, and tumor and immune cells display striking overexpression of PD-L1 and CD137 (TNFRSF9). In contrast, granulocytic MDSCs (g-MDSCs) are located farther from HRS cells and are more abundant in relapsed/refractory (R/R) patients [65]. These findings further support the idea that the biological impact of immune populations in cHL depends not only on their abundance, but also on their organization within the lesion.

**Figure 1:**
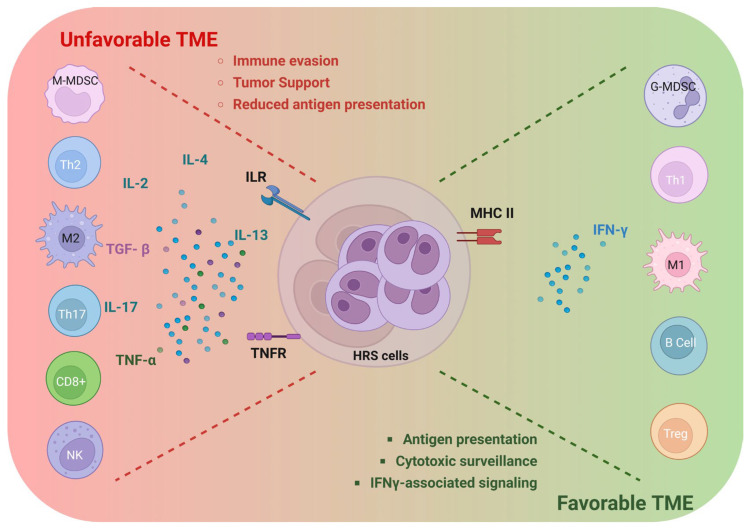
Conceptual model of favorable and unfavorable functional programs in the cHL microenvironment. HRS cells are embedded in a structured immune–stromal niche that spans tumor-supportive/immunosuppressive programs (left)—characterized by M2-like macrophages, Th2/Th17-associated cytokines, checkpoint-rich interactions (PD-L1 upregulation), and reduced antigen-presentation features—and comparatively more favorable immune contexts (right), enriched in Th1/cytotoxic and antigen-presenting programs, M1-like macrophages, and IFN-γ-associated signaling. The scheme summarizes representative cell types, cytokines, and membrane receptor interactions involved in proximity-dependent immune regulation in cHL. Created in BioRender, https://BioRender.com/bxpuff3, accessed on 13 April 2026.

Bulk transcriptomic and single-cell RNA sequencing studies later expanded these observations, revealing extensive heterogeneity among T-cell populations, including enrichment of helper, regulatory, and exhausted phenotypes [42,66,67,68,69]. These analyses also demonstrated immunosuppressive transcriptional programs in macrophages and highlighted contributions from fibroblasts and endothelial cells to extracellular matrix remodeling and cytokine signaling. However, such methods lacked spatial resolution and could not determine the spatial positions of distinct immune subsets relative to HRS cells or to one another. Consequently, whether specific macrophage or T-cell populations preferentially localize near tumor cells remained unresolved. Emerging spatial transcriptomic technologies now offer the opportunity to address these questions by integrating transcriptional state with tissue architecture, providing a more complete understanding of cellular organization and functional interactions within the immune microenvironment. A conceptual overview of favorable and unfavorable functional programs in the cHL microenvironment is shown in Figure 1.

## 4. Targeted Spatial Profiling of Immune Subsets in cHL

Complementary studies using targeted spatial profiling approaches have focused on selected immune populations and ROIs, providing clearer biological interpretation in clinically annotated cohorts and enabling hypothesis-driven dissection of tumor-adjacent versus immune-predominant compartments. In cHL, these efforts have particularly emphasized CD4^+^ T-cell programs, showing that functionally distinct CD4^+^ subsets occupy different spatial compartments within lesions. In GeoMx-based profiling, tumor-rich regions marked by CD30/PD-L1-high areas display transcriptional signatures consistent with T-cell activation coupled with functional impairment, whereas immune-predominant regions more distant from tumor cells exhibit alternative CD4^+^ states and distinct cytokine/chemokine milieus, with correlations to clinical metadata [70]. Targeted DSP has also been applied to CD4^+^ T-cell rosettes and their checkpoint landscape, supporting the concept that tumor-adjacent CD4^+^ structures are immunologically specialized microdomains rather than a uniform infiltrate [71]. Collectively, these data provide a compartmental framework (tumor-rich versus immune-predominant regions) that can be aligned with the spatially defined niches described by broader spatial transcriptomic and multimodal studies. Representative recent studies on the cHL microenvironment using spatial transcriptomics and other spatially resolved profiling approaches are summarized in Table 2.

This compartment-level view aligns with outcome-linked proximity features reported in relapsed/refractory (R/R) cHL. In particular, a proximity-based spatial prognostic model (RHL4S) identified PD-1^+^ CD4^+^ T cells as one of four spatial parameters that stratified post-ASCT outcomes, supporting the clinical relevance of quantifying PD-1^+^ CD4^+^ T cells within tumor-rich HRS niches using HRS-nearest-neighbor metrics [68]. More broadly, spatially defined tumor-rich compartments in cHL are repeatedly observed to be myeloid- and checkpoint-enriched, and CD4^+^ T-cell compartmentalization is consistent with this suppressive context even in studies not explicitly designed for outcome prediction. For example, integrative spatial profiling of the mononuclear phagocyte network showed that HRS-adjacent regions are enriched for classical monocytes, macrophages, and cDC2, with these populations expressing PD-L1, TIM-3, and IDO; enrichment of the cDC2–monocyte–macrophage network at diagnosis was associated with early treatment failure, consistent with a microenvironmental “pressure” that may shape which CD4^+^ subsets accumulate and/or are retained in tumor-proximal zones [69]. In refractory cHL, multiplex spatial analysis further highlighted topographic enrichment of suppressive myeloid features and candidate immune-evasion circuits, including CD137 and m-MDSCs in tumor-adjacent regions, with g-MDSCs preferentially located farther from HRS cells and enriched in non-responders, emphasizing that the prognostic impact of immune subsets is strongly spatially constrained [65].

Targeted immune profiling also aligns with cHL neighborhood models where malignant-cell states co-segregate with distinct immune architectures. Spatially resolved multiplexed profiling of HRS cell neighborhoods linked HRS MHC-I expression to immune-inflamed neighborhoods and identified HRS clustering (including syncytial-like patterns) associated with T-cell-excluded neighborhoods in subsets of EBV-negative tumors, consistent with the notion that CD4^+^ patterns in tumor-rich compartments may further segregate across excluded, Treg-high, or inflamed ecosystems [27]. These targeted observations are also consistent with genome-scale spatial mapping studies showing that malignant-cell neighborhoods are often helper T-cell-enriched and implicating IL-13 as a microenvironment-derived survival factor for HRS cells, providing a transcriptomic rationale for functionally relevant CD4-rich, tumor-adjacent niches [73]. Finally, relapse biology has begun to be framed in explicitly spatial terms: an early-relapse-associated suppressive circuit has been described in which LGALS9^+^ naïve B cells localize in close proximity to TIM-3^+^ CD4^+^ T cells and HRS cells, reinforcing that the key biological feature can be the location of specific CD4^+^ states relative to tumor cells and accessory populations, rather than their global abundance [42]. Altogether, these targeted and proximity-aware studies support a unifying concept: in cHL, where immune subsets reside is as important as which subsets are present, and tumor-adjacent microenvironments are the relevant units for mechanistic inference and biomarker translation.

## 5. Genome-Scale Spatial Mapping of the cHL Microenvironment

Recent genome-scale spatial transcriptomic studies have provided the first comprehensive maps of the cHL microenvironment in situ. These analyses revealed that HRS cells reside within distinct spatial neighborhoods enriched for specific immune and stromal cell types. Rather than being randomly distributed, CD4+ helper T cells, myeloid cells, and stromal elements assemble into organized niches surrounding HRS cells.

Notably, plasma cells and certain B-cell populations appear depleted from the immediate vicinity of HRS cells, suggesting spatial exclusion or selective recruitment mechanisms [73]. Ligand–receptor analyses performed in a spatially informed manner identified cytokine signaling pathways—most prominently the IL-13 axis—as potential survival signals for HRS cells. Functional validation in experimental systems demonstrated that disruption of IL-13 signaling impaired HRS cell viability, underscoring the biological relevance of spatially inferred interactions [73]. These findings are consistent with previous observations supporting the central role of the IL13/IL13R signaling pathway in cHL, including its proposed contribution to an autocrine growth loop for HRS cells and the rationale for therapeutic targeting of this pathway [74,75]. Taken together, these studies illustrate how genome-scale spatial mapping can move beyond descriptive tissue atlases to nominate biologically and potentially therapeutically relevant microenvironment-derived dependencies in cHL.

Complementary multimodal spatial studies further link HRS cell states (e.g., MHC-I expression and tumor-cell clustering) to immune-inflamed, T-cell-excluded, or Treg-high neighborhoods, highlighting that both malignant-cell programs and spatial context co-determine microenvironmental architecture [27].

## 6. System-Level Analysis of cHL Ecosystems

An essential step forward in understanding cHL will be the integration of tumor genomic lesions with spatial characterization of the TME, accounting for cellular dependencies and interactions. Such an approach may provide a more accurate pathophysiological representation of the disease with direct clinical implications. In this context, recent work by Aoki et al. [72] provides a comprehensive system-level analysis integrating HRS cell sequencing, spatial transcriptomics, and IMC. This study proposes four clinically relevant molecular subtypes (CST, CN913, STB, and CN2P) characterized by distinct architectural ecosystems in which malignant HRS cells are embedded.

HRS cells organize into subtype-specific niches enriched for particular immune partners—such as LAG3^+^ regulatory T cells, CXCL13^+^ helper T cells, or cytotoxic CD8^+^ T cells—forming spatially structured neighborhoods rather than random mixtures. Spatial transcriptomics and cell–cell interaction modeling further demonstrated that these niches correspond to unique signaling circuits (e.g., CXCL13–CXCR5 or PD-1–PD-L1 axes) that shape immune suppression or activation locally. Together, these spatial analyses show that clinically relevant cHL subgroups are defined by where immune cells are positioned relative to tumor cells and how they interact in situ, highlighting tumor architecture—not just cell types—as a key determinant of disease biology and potential therapeutic vulnerability.

Interestingly, this new proposal for genetic classification of cHL partially overlaps with two previously published ctDNA-based classifications. Thus, Aligh et al. [76] presented two genomic groups, H1 and H2, with the former exhibiting a higher mutational burden and the latter greater chromosomal instability. These characteristics largely overlap with the two groups proposed by Pirosa et al. [77], C1 and C2. In turn, the CST and STB subgroups share common characteristics with the H1/C1 groups, whereas CN913 and CN2P share common characteristics with H2/C2. Overall, the integration of tumor genomic lesions with spatial characterization of the TME adds an ecosystem-level dimension to existing molecular classifications and may improve patient stratification.

These ecosystem-level studies also help organize the major biological themes emerging from spatial analyses of cHL, which are discussed in the following section.

## 7. cHL-Specific Biological Themes Emerging from Spatial Studies

### 7.1. Spatial Niches and Tumor Support

A consistent theme across spatial studies is that HRS cells reside within specialized, non-randomly organized niches that provide trophic and immunomodulatory support. These tumor-adjacent neighborhoods are typically enriched for helper T-cell and myeloid populations, as well as stromal elements, and they can be comparatively depleted of plasma-cell compartments, suggesting selective recruitment and/or spatial exclusion near malignant cells [73].

**Figure 2:**
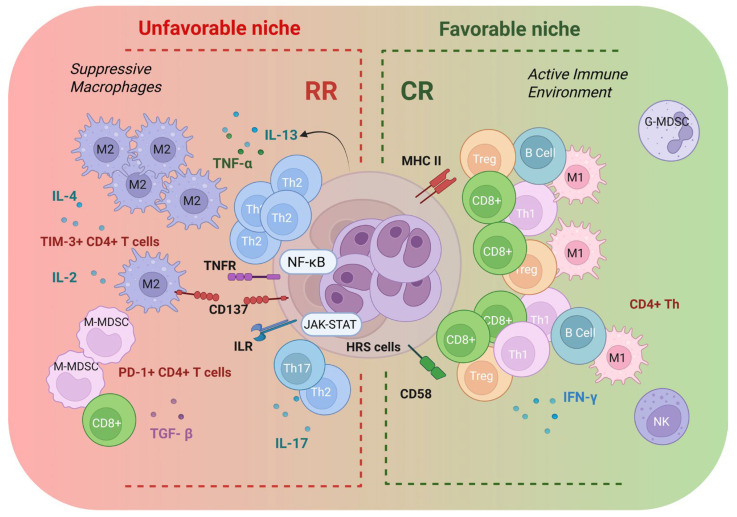
Representative spatial niches associated with complete response (CR) versus relapsed/refractory (R/R) cHL. Conceptual representation of tumor-proximal spatial niches described in cHL, contrasting comparatively favorable immune-inflamed architectures associated with CR and suppressive myeloid- and checkpoint-enriched niches associated with R/R disease. The scheme highlights representative cellular compositions, cytokine milieus, and receptor/signaling interactions observed in spatial studies of the cHL microenvironment. Created in BioRender, https://BioRender.com/bxpuff3, accessed on 13 April 2026.

Spatial inference has further nominated microenvironment-derived survival cues that are concentrated within these niches. In R/R settings, spatial architecture has also highlighted tumor-supportive crosstalk axes such as CXCR5+ HRS cell organization and their proximity to ligand-expressing macrophage populations, underscoring that niche biology can be therapeutically and prognostically relevant [68]. Representative CR-associated and R/R-associated spatial niches are schematically summarized in Figure 2.

### 7.2. Immune Evasion in Space

Spatial transcriptomics has clarified how immune evasion operates at short range in cHL. Immune checkpoint ligand expression and suppressive cytokine signaling are often enriched in cells immediately adjacent to HRS cells, suggesting that immune inhibition is locally concentrated rather than uniformly distributed throughout the tumor.

Relapse biology can also involve discrete suppressive circuits that are only apparent when spatial relationships are preserved. For example, early-relapse cHL has been associated with a niche in which LGALS9+ naïve B cells localized in proximity to TIM-3+ CD4+ T cells and HRS cells, consistent with spatially organized immune inhibition [42].

Single-cell transcriptomics coupled with spatial analysis in PD-1 blockade-treated cHL has revealed immune rewiring associated with resistance, including a circulating and tumor-infiltrating IL1β+ monocyte/macrophage program linked to lack of response [78]. Together, these observations support the view that immune evasion in cHL is spatially localized and ecosystem dependent.

### 7.3. Ligand–Receptor Interactions as Therapeutic Targets

By preserving spatial context, ligand–receptor analyses gain biological specificity, enabling candidate interactions to be interpreted within defined cellular neighborhoods where short-range signaling is most plausible. In cHL, IL-13/IL-4 receptor signaling has been highlighted as a microenvironment-derived survival pathway for HRS cells with supporting functional data, illustrating how spatial nomination can prioritize actionable axes [73].

Spatial and proximity-aware profiling has also emphasized chemokine-driven organization and potential therapeutic leverage points, including CXCL13–CXCR5-associated architecture in relapsed/refractory disease, which has been incorporated into clinically oriented spatial biomarker models [68].

In relapse-associated niches, the LGALS9–HAVCR2 (galectin-9–TIM-3) axis provides an additional example of a spatially constrained suppressive interaction that may not be evident from global frequencies alone [42].

Beyond cytokine- and chemokine-mediated interactions, microenvironment-informed single-cell analyses may identify additional therapeutically actionable immune-evasion circuits in cHL. Thus, recent in silico analysis of genomics and transcriptomics data highlights CD86 as a novel immunotherapeutic target [79]. The study builds on previously published genomics data, including microarray gene expression analysis of laser microdissected HRS cells, as well as bulk and single-cell transcriptomes of the TME in primary tumors. CD86 has been shown to be highly expressed not only by HRS cells but also by cHL-specific tumor-associated macrophages, while the CD86–CTLA-4 axis appears to contribute to T-cell exhaustion within the cHL microenvironment. Importantly, they next describe the development of CD86-28z CAR T cells, tested in vitro (HL-derived cell lines) and in vivo (cell-line xenotransplantation into NSG mice), to establish strong antigen-dependent CAR T-cell proliferation and effective T-cell-mediated killing of both malignant cells and CD86+ macrophages, supporting the concept that ecosystem-informed target discovery can be translated into microenvironment-directed therapeutic strategies in cHL [79].

Together, these studies support the view that spatially anchored ligand–receptor inference is most informative when coupled to neighborhood definitions and, where possible, orthogonal validation. Pathways such as IL-13/IL-4 receptor signaling, chemokine gradients, and checkpoint interactions can now be interpreted within defined cellular neighborhoods, improving prioritization of therapeutically actionable interactions.

Spatial analyses can also be integrated with subtype-specific genomic lesions to nominate additional signaling dependencies with therapeutic relevance. CSF2RB gene mutations, characteristic of the CST genetic subtype [72], were associated with dysregulated oncogenic signaling leading to ligand-independent constitutive activation of the JAK/STAT and PI3K/mTOR pathways. Notably, consistent with these observations, previous investigations examining JAK/STAT pathway inhibition in cHL identified CSF3R as a key molecule linked to hyperactive JAK/STAT signaling [80]. Pharmacological blockade of this pathway using ruxolitinib in cHL cellular models reduced malignant phenotypes, inducing apoptosis and reversing tumor-promoting inflammatory transcriptional programs. These findings highlight the functional importance of the G-CSF/CSFR signaling axis in HRS cells.

Early clinical evidence supports these observations. Ruxolitinib demonstrated measurable activity in preliminary clinical studies involving patients with advanced cHL [81,82]. In addition to direct antitumor effects, JAK inhibition has shown immunomodulatory properties. Specifically, ruxolitinib significantly reduced neutrophil-to-lymphocyte ratios and the proportion of myeloid-derived suppressor cells, while increasing cytokine-producing T cells. Furthermore, JAK inhibition restored the function of exhausted T cells and enhanced the efficacy of immune checkpoint blockade in preclinical solid tumor and lymphoma models [83]. These findings were recently corroborated in translational and clinical settings, demonstrating that JAK inhibition can potentiate checkpoint immunotherapy responses in Hodgkin lymphoma patients [80,83].

Collectively, these data support the rationale for prospective clinical trials designed to define the molecular context in which JAK/STAT inhibition may be most effective as a primary therapeutic strategy. In particular, combination approaches integrating JAK/STAT inhibitors with immunomodulatory agents or immune checkpoint inhibitors appear mechanistically justified and warrant systematic investigation.

## 8. Challenges and Future Directions

Spatially resolved characterization of the cHL microenvironment has important clinical implications. Spatial patterns may help explain heterogeneous responses to immune checkpoint inhibitors and identify biomarkers predictive of treatment outcomes. In the future, spatial transcriptomic signatures could inform risk stratification, guide combination therapies targeting both HRS cells and their supportive niches and refine patient selection for immunotherapies [84].

Spatial transcriptomics has ushered in a new era in the study of cHL, revealing that the TME is not only compositionally complex but also spatially organized into functional niches. These insights refine longstanding models of HRS cell dependency on the microenvironment and open new avenues for therapeutic intervention. As technologies mature and datasets expand, spatially resolved analyses are poised to become central to both biological discovery and clinical translation in cHL.

Despite rapid progress, several challenges remain. Current spatial transcriptomic platforms trade resolution for coverage, and integration across technologies remains computationally complex. Larger clinically annotated cohorts are needed to link spatial features with outcomes. Future directions include integration of spatial transcriptomics with spatial proteomics, epigenomics, and longitudinal sampling to capture dynamic microenvironmental changes during therapy.

At present, however, the remarkable capacity of spatial technologies to generate high-dimensional biological insight contrasts with the technical complexity, infrastructure requirements, and substantial costs that limit their widespread implementation in routine diagnostic workflows and standard clinical practice in the medium term. Nevertheless, their incorporation into academic research settings and prospective clinical trials is essential, particularly for the identification and validation of novel therapeutic targets in patients with refractory disease or unfavorable prognostic profiles. In the near term, spatial transcriptomics is likely to remain essential for biological discovery, biomarker identification, and therapeutic target prioritization, whereas translation into routine clinical decision-making will probably depend on the development of less complex surrogate approaches based on robust biomarkers and more accessible, standardized methodologies, such as IHC, multiplex immunofluorescence, and targeted NGS, capable of capturing the clinically actionable features of emerging molecular subtypes and tissue architectural ecosystems.

## Figures and Tables

**Table 1:** Practical comparison of representative spatial profiling platforms relevant to recent cHL studies (Visium, Slide-seq/Slide-seqV2, and CosMx).

Feature	Visium (10× Genomics)	Slide-seq/Slide-seqV2	Cosmx (Nanostring)
Method Type	Spatial sequencing with barcode capture spots	Spatial sequencing with barcoded micro-bead arrays	Multiplexed in situ imaging (FISH)
Commercial Availability	Fully commercial kit	No standard kit; academic protocol	Commercial product (instrument + panels)
Detection Principle	mRNA capture via barcodes → sequencing	mRNA captured on micro-beads with barcodes → sequencing	Fluorescent in situ detection of target molecules
Gene Coverage	Whole transcriptome	Whole transcriptome	Targeted panels. Whole-transcriptome assays (up to ~18,000 genes)
Spatial Resolution	~55 µm per spot (multiple cells per spot)	~10 µm per bead (near single-cell resolution)	Single-cell to subcellular resolution (<1 µm)
Detail Level	Groups of cells per spot	Nearly single-cell resolution	Single-molecule RNA/protein localization within cells
Data Type	Whole-transcriptome sequencing	Whole-transcriptome sequencing	Per-cell spatial counts derived from imaging (RNA ± protein)
Tissue Area Covered	Moderate (several regions of tissue)	Scalable (depends on bead array)	Variable, depends on the field of view and panel size
Sample Compatibility	Fresh frozen and FFPE (workflow-dependent)	Mostly fresh-frozen	Fresh frozen and FFPE
Strengths	Whole transcriptome; standardized commercial workflow	High cellular resolution; full transcriptome	Very high (subcellular) resolution; multiplexed RNA + protein
Limitations	Not single-cell; spots mix multiple cells	Not a commercial kit; requires technical expertise	Limited panels; whole transcriptome only recently available; complex analysis

**Table 2:** Representative recent studies (2020–2026) on the cHL microenvironment using spatial transcriptomics and complementary spatially resolved profiling approaches.

Study (Year; Journal)	Disease/Setting	Spatial Platform(s)	Key Microenvironment Finding Enabled by Spatial Context	N	Type of Study	Translational/Clinical Implications
Aoki et al. [72]	cHL	HRS cell sequencing, spatial transcriptomics and IMC	Spatial transcriptomics and cell–cell interaction models identify subtype-specific niches enriched for particular immune partners—such as LAG3^+^ Treg cells, CXCL13^+^ Th cells, or cytotoxic CD8^+^ T cells—forming spatially structured neighborhoods rather than random mixtures.	Targeted seq: 114 cases (frozen) WES: 6 cases tumor-normal paired TMAs containing 114 for IMC	Multidimensional characterization and comprehensive system-level analyses.	Proposal of four clinically relevant molecular subtypes (CST, CN913, STB, and CN2P). Mutation-driven mechanisms of deregulated cytokine signaling suggest new therapeutic interventions
Shanmugam et al. [73]	cHL	Genome-wide spatial + single-cell-resolved transcriptomics (integrated)	Defines a malignant-cell-centered niche and nominates IL13 as a microenvironment-derived tumor survival factor; integrates functional dependency evidence on IL4R/IL13RA.	SlideSeqV2: 12 cases vs. 7 RLN CosMx: 4 cases	Translational multi-platform study with functional validation.	Moves from descriptive spatial atlases to spatially nominated, actionable microenvironmental dependencies (supports testing IL13-axis therapies).
Stewart et al. [69]	cHL diagnostic biopsies vs. non-lymphoma LNs	Spatial transcriptomics + scRNA-seq + multiplex IF	High-resolution mapping of mononuclear phagocyte subsets and their spatial polarization relative to HRS cells; identifies immunoregulatory checkpoint expression in cDCs/monocytes and links a myeloid network to early treatment failure.	mIF: 54 cases GeoMX: 10 cases (Plus scRNAseq data from Aoki et al. 2020) [67]	Observational integrated spatial + single-cell tissue study with outcome association.	Prioritizes myeloid-centered spatial niches as biomarker/target space beyond tumor-cell PD-L1 alone.
Pourmaleki et al. [27]	Newly diagnosed EBV+/EBV− cHL	Multiplex spatial protein imaging + transcriptomic sequencing (neighborhood analysis)	Links HRS cell states (e.g., MHC-I; spatial clustering) to distinct immune neighborhoods (inflamed vs. excluded vs. Treg-high).	mIF analyses: 36 cases Bulk transcriptomics (Nanostring): 32 cases	Observational spatial profiling; hypothesis-generating.	Frames cHL as spatial immunophenotypes for immunotherapy biomarker hypotheses.
Aoki et al. [68]	Relapsed/refractory cHL; post-ASCT risk	Imaging mass cytometry; validation by multicolor IF	Uses proximity-based metrics to define relapse ecosystems and derives a reduced spatial-score prognostic assay.	71 cases, paired diagnosis and relapse, plus 22 cases CR	Retrospective biomarker development with independent validation.	Demonstrates a pragmatic route from discovery spatial profiling to deployable pathology assays for risk stratification.
Menéndez et al. [70]	cHL; compartmental (tumor-rich vs. immune-predominant)	Spatially resolved profiling of CD4^+^ T-cell architecture	Maps CD4^+^ T-cell variation across intratumoral compartments and correlates with clinicopathologic features.	24 cases (12 R/R vs. 12 CR cases)	Observational spatial pathology study.	Adds a pathology-facing compartment framework to interpret cHL immune topography (biomarker hypothesis generation).
Yin et al. [42]	cHL diagnostic vs. relapse (paired)	IMC (spatial) + scRNA-seq	Spatially confirms a relapse-associated niche featuring LGALS9^+^ naïve B cells near TIM-3^+^ CD4^+^ T cells and HRS cells. Enrichment in naïve B-cells in early relapse tumors	Discovery cohort: 8 patients (16 samples, paired diagnostic/relapse) Validation cohort 25 samples	Paired-sample observational multi-omic study with spatial validation.	Nominates a relapse-specific Galectin-9/TIM-3 immunoregulatory axis as biomarker/target hypothesis.
Solórzano et al. [65]	cHL	Spatially resolved profiling (multiplex IF)/computational spatial biomarker framing	Provides generalizable concepts for spatial biomarker development/validation (e.g., compositional vs. proximity metrics).	30 cases Validation cohort: 130 samples	Observational spatial pathology study (conceptual/methods context; not a cHL spatial transcriptomics primary study).	Useful to justify why spatial context adds value and what is required for clinical translation in HL (prospective validation; reproducibility).
Aoki et al. [67]	cHL	scRNA-seq + multiplex IF	Provides generalizable concepts for spatial biomarker analyses, proximity metrics,…	22 cases + 5 RLN	Observational spatial pathology study (conceptual/methods context; not a cHL spatial transcriptomics primary study).	LAG3+ T-cells in the direct vicinity of MHC-II-deficient HRS cells

## Data Availability

No new data were created or analyzed in this study. Data sharing is not applicable to this article.

## Abbreviations

The following abbreviations are used in this manuscript:

ASCT	Autologous stem cell transplantation
C1	ctDNA-based genomic cluster (C1)
C2	ctDNA-based genomic cluster (C2)
CCL17	C-C motif chemokine ligand 17 (TARC)
CCL22	C-C motif chemokine ligand 22
CCL5	C-C motif chemokine ligand 5
CD4	CD4 T cells
CD8	CD8 T cells
CD137	4-1BB (gene: TNFRSF9)
CD137L	4-1BB ligand
cDC2	Conventional dendritic cell type 2
cHL	Classical Hodgkin lymphoma
CN2P	cHL molecular subtype code (Aoki et al.)
CN913	cHL molecular subtype code (Aoki et al.)
CosMx	NanoString CosMx Spatial Molecular Imaging
CR	Complete response
CSF-1	Colony-stimulating factor 1
CSF2RB	Colony-stimulating factor 2 receptor subunit beta
CSF3R	Colony-stimulating factor 3 receptor
CST	cHL molecular subtype code (Aoki et al.)
CtDNA	Circulating tumor DNA
CTLA-4	Cytotoxic T-lymphocyte–associated protein 4
CXCL13	C-X-C motif chemokine ligand 13
CXCR5	C-X-C chemokine receptor 5
DestVI	Destination-Variational Inference (deconvolution method)
DSP	Digital Spatial Profiling (NanoString GeoMx)
EBV	Epstein–Barr virus
FFPE	Formalin-fixed, paraffin-embedded
FISH	Fluorescence in situ hybridization
G-CSF	Granulocyte colony-stimulating factor
g-MDSC	Granulocytic myeloid-derived suppressor cell
H1	ctDNA-based genomic group (H1)
H2	ctDNA-based genomic group (H2)
HAVCR2	Gene symbol for TIM-3
HDST	High-definition spatial transcriptomics
HRS	Hodgkin and Reed–Sternberg (cells)
IHC	Immunohistochemistry
IMC	Imaging mass cytometry
IDO	Indoleamine 2,3-dioxygenase
IFN-γ	Interferon gamma
IL-4	Interleukin 4
IL-10	Interleukin 10
IL-13	Interleukin 13
IL-13R	Interleukin 13 receptor
ISH	In situ hybridization
JAK	Janus kinase
JAK/STAT	Janus kinase/signal transducer and activator of transcription pathway
LAG-3	Lymphocyte activation gene 3
MDSC	Myeloid-derived suppressor cell
m-MDSC	Monocytic MDSC
mIF	Multiplex immunofluorescence
MHC	Major histocompatibility complex
MHC-I	Major histocompatibility complex class I
MHC-II	Major histocompatibility complex class II
MERFISH	Multiplexed error-robust fluorescence in situ hybridization
mTOR	Mechanistic target of rapamycin
NGS	Next-generation sequencing
NK	Natural killer (cells)
PD-1	Programmed cell death protein 1
PD-L1	Programmed death-ligand 1
PI3K	Phosphoinositide 3-kinase
R/R	Relapsed/refractory
RCTD	Robust cell type decomposition (deconvolution method)
RHL4S	Proximity-based spatial prognostic model (name used in cited study)
ROI	Region of interest
scRNA-seq	Single-cell RNA sequencing
seqFISH	Sequential fluorescence in situ hybridization
STAT	Signal transducer and activator of transcription
STB	cHL molecular subtype code (Aoki et al.)
TARC	Thymus and activation-regulated chemokine (CCL17)
TGF-β	Transforming growth factor beta
Th1	T helper 1
Th2	T helper 2
Th17	T helper 17
TIGIT	T-cell immunoreceptor with Ig and ITIM domains
TIM-3	T-cell immunoglobulin and mucin-domain containing-3
TME	Tumor microenvironment
TNFRSF9	Tumor necrosis factor receptor superfamily member 9 (CD137)
Tregs	Regulatory T cells
WTA	Whole-transcriptome approach/Whole Transcriptome Atlas (GeoMx WTA)
